# Vitamin D Levels and the Risk of Posttransplant Diabetes Mellitus After Kidney Transplantation

**DOI:** 10.1177/15269248211002796

**Published:** 2021-04-01

**Authors:** Kevin Quach, Monica Abdelmasih, Pei Xuan Chen, Yanhong Li, Olusegun Famure, Michelle Nash, Ramesh Prasad, Bruce A. Perkins, Paul M. Yip, S. Joseph Kim

**Affiliations:** 1 Division of Nephrology and the Kidney Transplant Program, 7989 University Health Network, Toronto, Ontario, Canada; 2 Division of Nephrology, St. Michael’s Hospital and 7938 University of Toronto, Toronto, Ontario, Canada; 3 Division of Endocrinology, Universal Health Network and 7938 University of Toronto, Toronto, Ontario, Canada; 4 Laboratory Medicine and Pathobiology, University Health Network and 7938 University of Toronto, Toronto, Ontario, Canada; 5 Division of Clinical Biochemistry, 7938 University of Toronto, Toronto, Ontario, Canada; 6 Institute of Health Policy, Management and Evaluation, 7938 University of Toronto, Toronto, Ontario, Canada

**Keywords:** kidney transplantation, vitamin d, post-transplant diabetes mellitus

## Abstract

**Introduction:**

Given the burden of posttransplant diabetes mellitus and the high prevalence of low vitamin D levels in kidney transplant recipients, it is reasonable to consider vitamin D as a novel and potentially modifiable risk factor in this patient population.

**Research question:**

To determine the association between 25- hydroxyvitamin D (25(OH)D) level and posttransplant diabetes among kidney transplant recipients. Design: In a multi-center cohort study of 442 patients who received a kidney transplant between January 1, 2005 and December 31, 2010, serum samples within one-year before transplant were analyzed for 25(OH)D levels. The association between 25(OH)D and posttransplant diabetes were examined in Cox proportional hazard models.

**Results:**

The median 25(OH)D level was 66 nmol/L. The cumulative probability of diabetes at 12-months by quartiles of 25(OH)D (< 42, 42 to 64.9, 65 to 94.9, and > 95 nmol/L) were 23.4%, 26.9%, 21.4%, and 15.6%, respectively. Compared to the highest 25(OH)D quartile, hazard ratios (95% CI) for the risk were 1.85 (1.03, 3.32), 2.01 (1.12, 3.60), 1.77 (0.96, 3.25) across the first to third quartiles, respectively. The associations were accentuated in a model restricted to patients on tacrolimus. When modeled as a continuous variable, 25(OH)D levels were significantly associated with a higher risk of diabetes (hazard ratio 1.06, 95% CI: 1.01, 1.13 per 10 nmol/L decrease).

**Discussion:**

Serum 25(OH)D was an independent predictor of posttransplant diabetes in kidney transplant recipients. These results may inform the design of trials using vitamin D to reduce the risk in kidney transplant recipients.

## Introduction

Kidney transplantation is the gold standard for the treatment of end-stage kidney disease (ESKD).^1^ Posttransplant diabetes mellitus (PTDM) after kidney transplantation has emerged as an important complication after kidney transplantation leading to increased health expenditures in recipients.^2^ The time between the diagnosis of diabetes and the development of diabetic complications was shorter in these patients.^2^ Not only was the burden of PTDM high in kidney recipients, it was also associated with inferior graft and patient outcomes, such as graft failure, death-censored graft failure, and mortality.^2^ Patients with PTDM were at an approximately 3-fold increased risk of cardiac death or nonfatal myocardial infarction.^2^ The reported cumulative incidence of PTDM in an US-based study was estimated to be 9.1%, 16.0%, and 24.0% at 3, 12, and 36-months post-transplant, respectively.^3^ Common risk factors for PTDM include older age, black race, higher body mass index, and use of calcineurin inhibitors (CNI).^4^ More recently, vitamin D insufficiency and deficiency have also been suggested to increase the risk of PTDM in kidney transplant recipients.

Vitamin D is a hormone with protean effects in human beings. Apart from its traditionally recognized role in regulating calcium/phosphorus metabolism and skeletal bone health, there is mounting evidence for its role in the maintenance of general health and well-being.^5^ Both experimental and observational studies have indicated that lower levels of vitamin D are associated with an increased risk of cancer, cardiovascular disease, multiple sclerosis, depression, cognitive decline, and diabetes mellitus.^5^

Abnormal vitamin D physiology is a central feature of ESKD, since the kidneys play a major role in the conversion of vitamin D to its biologically active form.^6^ Consequently, there is a high prevalence of hypovitaminosis D among patients with ESKD. It is estimated that 89 to 96% of ESKD patients on dialysis are either vitamin D insufficiency or deficient.^6^ In addition, within the kidney transplant recipient population, genetic polymorphisms of TaqI^7^ and Fok1^8^ allele in the vitamin D receptor are identified as significant risk factors for PTDM.

### Specific Aims

In light of the significant burden of PTDM and the high prevalence of low vitamin D levels in kidney recipients, it is reasonable to consider vitamin D as a novel and potentially modifiable risk factor for PTDM in this patient population. Moreover, established risk factors for PTDM in kidney recipients have been strongly associated with low vitamin D levels. This further lends credence to the hypothesis that vitamin D is an important determinant of PTDM risk. Considering the compelling data on the relationship between vitamin D and PTDM in the laboratory and in human population, in addition to the paucity of literature on this topic, our study aims to study the link between vitamin D status and the risk of PTDM development in kidney recipients. This may provide new insights into the potential use of vitamin D as a preventive for PTDM.

## Methods

### Design and Setting

We conducted an observational cohort study at University Health Network (UHN) and St. Michael’s Hospital (SMH) in Toronto, Ontario. Approval was obtained from the Research Ethics Boards of both institutions.

### Population and Sample

We included all adult kidney transplant recipients who received a primary living or deceased donor kidney at either institution between January 1, 2005 and December 31, 2010. Exclusion criteria included (*a*) prior non-kidney transplant, (*b*) transplants outside of UHN or SMH, (*c*) re-grafts, (*d*) desensitization prior to transplant, (*e*) primary non-function, and (*f*) history of diabetes mellitus at time of transplant. These exclusion criteria were chosen and applied a priori. Patients were followed until they developed PTDM, or until the time of graft loss, death, or until December 31, 2011.

### Data Collection

All patient data were obtained from the Comprehensive Renal Transplant Research Information System,^9^ hospital electronic patient record systems, and patient charts. The following data were collected and included in statistical analyses: (1) recipient age, gender, race, body mass index, time on dialysis, cause of ESKD, (2) donor type, and (3) type of induction, prednisone at discharge, type of CNI, albumin, calcium and parathyroid hormone levels, transplant season, and transplant era. The seasons were defined as follows: fall (September 21 to December 20), winter (December 21 to March 20), spring (March 21 to June 20), and summer (June 21 to September 20). Among the UHN cohort only, recipient socioeconomic status was estimated by mapping recipient postal codes to median household income using the 2006 Canadian census data.

Accessible patient serum samples closest to the time of transplantation were retrieved from the histocompatibility laboratory (HLA lab) for the measurement of 25-hydroxyvitamin D [25(OH)D], intact parathyroid hormone (PTH), albumin, calcium, and phosphorus. Levels of 25(OH)D were measured using high performance liquid chromatography (Transcend™ TLX-2 system, ThermoScientific) coupled to a mass spectrometer (API 5000, AB Sciex). Intact PTH was measured by chemiluminescent immunoassay (Immulite 2000, Siemens Medical Solutions). Albumin, calcium, and phosphorus were measured on the ARCHITECT chemistry analyzer (Abbott Diagnostics).

### Exposure and Outcome Classification and Assessment

The primary exposure was the serum level of 25(OH)D within one year before kidney transplantation. The major circulating form of vitamin D is 25(OH)D and is a more reliable measure of the body’s vitamin D stores. Furthermore, the measurement of 1,25-dihydroxyvitamin D [1,25(OH) _2_ D] is more cumbersome, associated with greater variability, and has a shorter half-life compared to 25(OH)D. The National Kidney Foundation Kidney Disease Outcomes Quality Initiative (KDOQI) for patients with chronic kidney disease (i.e., replete > 75 nmol/L, insufficiency 40 to 75 nmol/L, and deficiency < 40 nmol/L) was used to divide 25(OH)D into four clinically meaningful groups. The replete or normal category served as the referent group.

The outcome of interest was PTDM as defined according to the American Diabetes Association criteria: (*a*) fasting plasma glucose ≥ 7.0 mmol/L, (*b*) casual glucose ≥ 11.1 mmol/L with symptoms of diabetes, or (*c*) two-hour plasma glucose ≥ 11.1 mmol/L during a 75 g oral glucose tolerance test. The diagnosis of PTDM required at least two abnormal plasma glucose measurements on two separate occasions. The date of the first elevated plasma glucose was considered the index date for PTDM. A single research assistant without knowledge of 25(OH)D status ascertained all PTDM events.

### Data Analysis

Differences in the distribution of baseline characteristics across vitamin D groups were examined using the Student’s t-test or the Wilcoxon rank sum test for continuous data and the chi-square test or the Fisher’s exact test for categorical data. Time to PTDM as a function of 25(OH)D levels was graphically assessed using the Kaplan-Meier product limit method, and differences across survival functions were ascertained using the log rank test. Multivariable Cox proportional hazards models were fitted to determine the independent association of 25(OH)D levels and PTDM adjusting for recipient, donor, and transplant factors. Acute rejection episodes were modeled as a time-varying covariate in the Cox proportional hazards model.

In order to detect potential nonlinear effects, 25(OH)D was modeled both as a categorical and continuous variable. The Fine and Gray modification of the Cox model was also fitted to formally account for death and death-censored graft failure as competing events to PTDM. Schoenfeld residuals and log (cumulative hazard) curves were used to examine the proportionality assumption (no violations from proportionality were observed). To evaluate the presence of interaction of vitamin D and PTDM by recipient age, sex, race, cause of ESKD, and CNI type, likelihood ratio testing was performed to assess the statistical significance of interaction terms. Multiple imputation was used to assign missing covariate data.

All statistical analyses were performed using Stata/MP version 12.1 (College Station, TX). A two-tailed *P* value of < 0.05 was considered statistically significant. The research ethics board of the University Health Network approved the study. The STROBE guidelines were used to organize the manuscript.

### Sensitivity Analysis

The impact of analytical assumptions on the main results were explored in the following sensitivity analyses: (*a*) modeling 25(OH)D as a continuous variable; (*b*) re-categorizing 25(OH)D levels into quartiles to ensure sufficient numbers of events per group for statistical modeling; (*c*) restricting the analysis to PTDM cases occurring in the first 6 and 12 months posttransplant since this may be a more etiologically relevant time period to assess the impact of baseline 25(OH)D levels on the risk of PTDM; and (*d*) limiting the analysis to patients whose 25(OH)D levels were ascertained from samples taken within 3 months prior to the day of transplantation.

## Results

**Figure 1.**
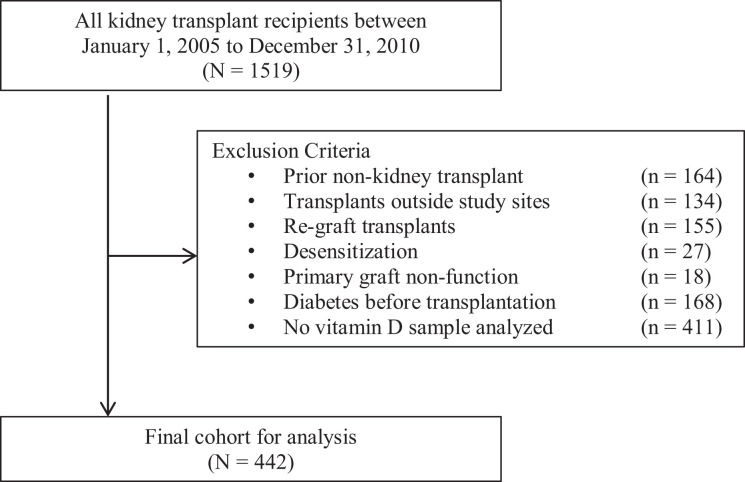
Study flow diagram.

After applying the exclusion criteria, 442 patients with analyzed vitamin D samples comprised the final study cohort (Figure 1). There were 411 patients who had insufficient samples for measurement of 25(OH)D. In total, there were 124 patients who developed PTDM over a follow-up of 823.95 person years. In the first 6 and 12-months posttransplant, 86 and 96 patients were diagnosed with PTDM, respectively.

Table 1 summarizes the baseline recipient, donor, and transplant characteristics of transplant recipients by 25(OH)D strata. Male gender, white race, shorter time on dialysis, high albumin levels, and living donor transplants were all associated with higher 25(OH)D levels. Among transplant characteristics, kidney transplants in 2009 to 2010 and use of an IL2-receptor blocker for induction therapy were associated with higher 25(OH)D levels. Tacrolimus was the most commonly used CNI during the study period. Serum samples taken in the summer and fall were also associated with higher 25(OH)D levels. When compared to the total eligible population (N = 1519), the study cohort had a lower proportion of males (55.7% vs. 62.6%), longer median time on dialysis (4.9 vs. 3.7 years), and a lower proportion treated with depleting induction therapy (52.8% vs. 60.7%).

**Table 1.** Study Characteristics by Vitamin D Strata.

Study characteristics	Total (N = 442)	Vitamin D groups			
		*Deficiency:* < 40.0 nmoL/L (n = 97)	*Insufficiency:* 40.0 -74.9 nmoL/L (n = 166)	*Replete:* > 75.0 nmoL/L (n = 179)	
	N (%)	N (%)	N (%)	N (%)	*P* value
Recipient Characteristics					
Recipient male sex	55.7%	45.4%	51.2%	65.4%	
Recipient non-White (vs. White) race	37.7%	66.3%	31.8%	29.0%	
Cause of End-stage Renal Disease					0.20
Glomerulonephritis	48.0%	50.5%	48.8%	45.8%	
Polycystic kidney disease	15.6%	9.3%	14.5%	20.1%	
Other	36.4%	40.2%	36.8%	34.1%	
	Mean (SD)	Mean (SD)	Mean (SD)	Mean (SD)	
Recipient age at transplant (years, mean [SD])	50.8 (13.8)	50.9 (14.1)	50.9 (14.2)	50.7 (13.3)	0.99
Recipient Body Mass Index (Kg/m^2^, mean [SD])	25.9 (5.0)	25.1 (4.7)	26.6 (5.4)	25.8 (4.7)	0.06
Albumin (g/L, mean [SD])	41.5 (4.6)	38.9 (4.6)	41.7 (4.3)	42.7 (4.3)	< 0.001
Calcium (mmol/L, mean [SD])	2.5 (0.4)	2.5 (0.7)	2.4 (0.3)	2.5 (0.3)	0.89
	Median [IRQ]	Median [IRQ]	Median [IRQ]	Median [IRQ]	
Time on dialysis (years, median [IQR])	4.9 (2.2, 7.0)	5.9 (2.7, 7.5)	4.6 (1.7, 7.1)	4.5 (2.1, 6.7)	0.01
Parathyroid hormone (pmol/L, median [IQR])	25.1 (11.4, 56.7)	27.0 (12.6, 58.1)	23.7 (13.4, 63.5)	25.5 (9.8, 45.9)	0.56
Donor Characteristics	N (%)	N (%)	N (%)	N (%)	
Deceased (vs. living) donor	58.8%	70.1%	56.6%	54.8%	0.04
Transplant Characteristics					
Depleting (vs. non-depleting) induction therapy	52.8%	59.0%	57.3%	45.5%	0.04
Tacrolimus (vs. cyclosporine)	81.9%	81.1%	81.1%	83.1%	0.87
Prednisone at discharge	95.0%	94.9%	96.4%	93.8%	0.55
University Health Network (vs. St. Michael’s Hospital)	52.3%	45.4%	57.8%	50.8%	0.13
Season					< 0.001
Spring	18.3%	19.6%	26.5%	10.1%	
Summer	21.0%	13.4%	16.3%	29.6%	
Fall	41.6%	41.2%	34.9%	48.0%	
Winter	19.0%	25.8%	22.3%	12.3%	
Transplant era					0.02
2005 – 2008	34.8%	37.1%	31.9%	36.3%	
2009 – 2010	33.5%	42.3%	36.1%	26.3%	

**Figure 2.**
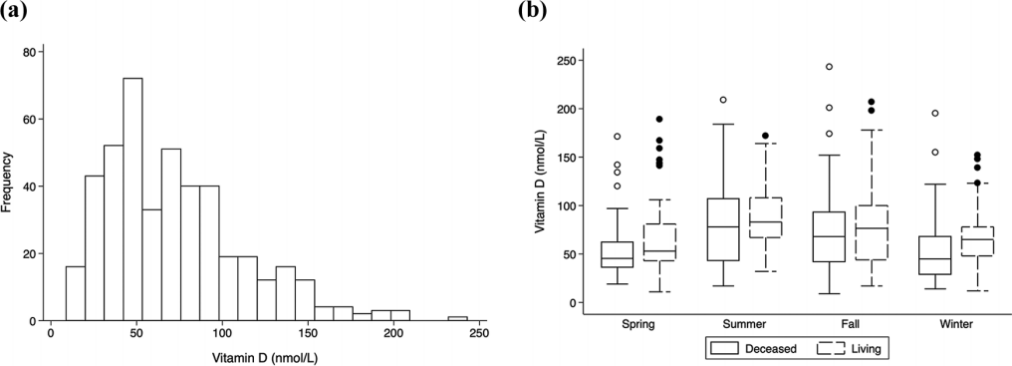
Distribution of Baseline Vitamin D Levels by Season (Left) And Donor Type (Right).

Figure 2 shows the distribution of 25(OH)D levels, stratified by season of serum sample and donor type. The distribution of 25(OH)D levels was right-skewed and ranged from 9 to 243 nmol/L, with a median (interquartile range) of 66 (51) nmol/L and a mean (SD) of 72.4 (40.6) nmol/L. One quarter of the observations were below 37.5 nmol/L and one quarter of observations were above 92 nmol/L. When stratified by donor type, median 25(OH)D levels were lower among deceased donor recipients compared to living donor recipients (58 vs. 71 nmol/L). When stratified by season, median 25(OH)D levels were highest during the summer (80 nmol/L), followed by fall (72.5 nmol/L), winter (50 nmol/L), and spring (49 nmol/L). Living donor recipients had higher 25(OH)D levels across all seasons.

**Figure 3.**
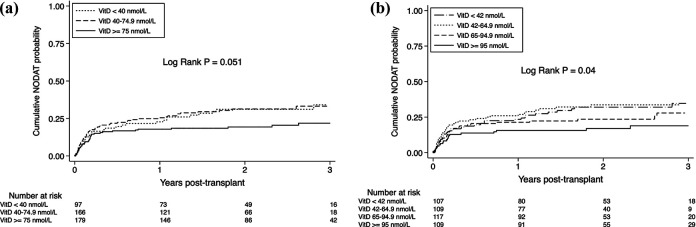
Kaplan-Meier Curves, Stratified by Vitamin D Levels, for the Cumulative Probability of Posttransplant Diabetes Using NFK-KDOQI Categories (Top) And Quartiles (bottom).

Figure 3 depicts the cumulative probability of developing PTDM stratified by 25(OH)D category. The highest 25(OH)D group, which was determined using the KDOQI categories, showed a lower risk of PTDM throughout the post-transplant follow-up (log-rank *P*=0.051). The cumulative probability of PTDM at 12-months in the < 40, 40 to 74.9, and the ≥ 75 nmol/L 25(OH)D groups were 22.7%, 25.5%, and 17.9%, respectively. When examined as quartiles, the highest 25(OH)D group showed a reduced risk for PTDM (log-rank *P* = 0.04). The cumulative probability of PTDM at 12-months in the < 42, 42 to 64.9, 65 to 94.9, and > 95 nmol/L groups were 23.4%, 26.9%, 21.4%, and 15.6%, respectively.

Table 2 shows the results of the multivariable Cox proportional hazards models for the association of 25(OH)D and the relative hazard of PTDM over follow-up. When assessed as a continuous variable, a 10 nmol/L decrease in 25(OH)D was associated with a significantly increased risk of PTDM (hazards ratio [HR] 1.06, 95% confidence interval [CI] 1.01, 1.13). When using KDOQI categories, the hazard ratios (95% CI) for the risk of PTDM were 1.52 (0.96, 2.39) and 1.30 (0.76, 2.23) for the 25(OH)D deficiency group (< 40.0 nmol/L) and the vitamin D insufficiency group (40.0 to 74.9 nmol/L), respectively, when compared to the vitamin D sufficient group (≥ 75 nmol/L). When 25(OH)D was modeled using quartiles, the hazard ratios (95% CI) for the risk of PTDM were 1.85 (1.03, 3.32), 2.01 (1.12, 3.60), 1.77 (0.96, 3.25) for 25(OH)D groups < 42.0, 42.0 to 64.9, and 65.0 to 94.9 nmol/L, respectively.

**Table 2.** Association of Baseline Vitamin D Levels and the Risk of Post-Transplant Diabetes Mellitus Using Multivariable Cox Proportional Hazards Models.

Vitamin D (nmoL/L)	Hazard Ratio (95% CI) of PTDM			
	Full study cohort	*P* value	Patients on Tacrolimus	*P* value
Continuous (per 10 nmol/L decrease)	1.06 (1.01, 1.13)	0.03	1.09 (1.02, 1.16)	0.01
Categorical				
NKF-KDOQI				
Replete: >75.0	referent		referent	
Insufficient: 40.0-74.9	1.52 (0.96, 2.39)	0.07	1.96 (1.16, 3.31)	0.01
Deficient: <40.0	1.30 (0.76, 2.23)	0.34	1.72 (0.93, 3.20)	0.08
Statistical quartile				
>95.0	referent		referent	
65.0-94.9	1.85 (1.03, 3.32)	0.04	2.09 (1.07, 4.10)	0.03
42.0-64.9	2.01 (1.12, 3.60)	0.02	2.71 (1.39, 5.30)	0.004
<42.0	1.77 (0.96, 3.25)	0.07	2.16 (1.07, 4.36)	0.03

Abbreviations: NFK-KDOQI, National Kidney Foundation Kidney Disease Outcomes Quality Initiative.

A subsequent model to assess the effect of recipient socioeconomic status and acute rejection as a time-varying covariate in the subcohort with this data did not show important deviations from the original effect estimates. Furthermore, excluding PTDM events in the first 1- or 3-months post-transplant showed similar results to the main analysis (Supplemental Table 1). Since approximately 82% of patients was treatment with tacrolimus, adjustment for the type of calcineurin inhibitor may contribute to model instability. A model restricted to patients treated with tacrolimus showed an accentuation of the associations observed in the primary model (Table 2). All model coefficients and the degree of missingness for model covariates are shown in Supplemental Tables 2 and 3.

Sensitivity analyses were conducted to test the robustness of the primary results are shown in Table 3. In fully adjusted multivariable Cox proportional hazards models, the association between 25(OH)D and PTDM at 6 and 12-months was not significant when 25(OH)D was considered as a continuous variable. When modeled as a categorical variable using quartiles, vitamin D was a significantly associated with PTDM at 6 months, but not at 12 months. All analyses showed that lower vitamin D levels were associated with higher risk of PTDM, though only some showed a statistically significant difference. A subsequent analysis restricted the cohort to patients from whom serum samples within three months prior to transplant were available. In this analysis, there was a stronger association between lower 25(OH)D and the risk of PTDM, compared with the results from the main analysis. Finally, to address the possibility that graft failure and death were informative censoring events, a competing risk Cox proportional hazards model was fitted. When 25(OH)D was modeled as a continuous variable, the analysis demonstrated the results were unaltered from the primary results. Similarly, the competing analysis demonstrated that 25(OH)D as a quartile was significantly associated with PTDM at all levels in the competing risk model.

**Table 3.** Sensitivity Analyses of the Association Between Vitamin D Levels and Post-Transplant Diabetes Mellitus.

Vitamin D (nmoL/L)	Hazard ratio (95% C.I.) of PTDM							
	PTDM within 6 months posttransplant	*P*	PTDM within 12 months posttransplant	*P*	Sample within 3 months prior to transplant	*P*	Accounting for death and graft failure as competing events	*P*
Continuous (per 10 nmol/L decrease)	1.03 (0.97, 1.11)	0.32	1.05 (0.98, 1.12)	0.17	1.06 (0.99, 1.14)	0.08	1.06 (1.00, 1.13)	0.03
Categorical								
NKF-KDOQI								
Replete: >75.0	referent		referent		referent		referent	
Insufficient: 40.0-74.9	1.42 (0.84, 2.40)	0.19	1.48 (0.90, 2.45)	0.13	1.47 (0.82, 2.63)	0.20	1.50 (0.92, 2.44)	0.10
Deficient: <40.0	1.08 (0.56, 2.08)	0.83	1.18 (0.64, 2.20)	0.59	1.33 (0.67, 2.64)	0.42	1.30 (0.76, 2.23)	0.33
Statistical quartile								
>95.0	referent		referent		referent		referent	
65.0-94.9	1.93 (0.98, 3.79)	0.06	1.81 (0.95, 3.44)	0.07	2.48 (1.17, 5.25)	0.02	1.88 (1.04, 3.39)	0.04
42.0-64.9	1.91 (0.97, 3.77)	0.06	1.92 (1.02, 3.64)	0.05	2.34 (1.14, 4.83)	0.02	2.11 (1.11, 3.99)	0.02
<42.0	1.49 (0.71, 3.12)	0.29	1.60 (0.80, 3.19)	0.18	1.96 (0.92, 4.18)	0.08	1.80 (0.97, 3.33)	0.06

Abbreviations: NKF-KDOQI, National Kidney Foundation Kidney Disease Outcomes Quality Initiative; PTDM, posttransplant diabetes mellitus.

To examine any potential effect measure modification by previously determined patient characteristics, interaction terms were included in the Cox model. The interaction terms between 25(OH)D and recipient age (*P* = 0.32), gender (*P* = 0.34), race (*P* = 0.55), cause of ESKD (*P* = 0.89), and CNI type (*P* = 0.33) were not statistically significant, as indicated by the likelihood ratio test.

## Discussion

In our cohort of 442 Canadian kidney transplant patients, recipient serum 25(OH)D was inversely associated with PTDM, independent of recipient, donor, and transplant characteristics. When 25(OH)D was modeled as a categorical or continuous variable, lower 25(OH)D was associated with a higher risk of PTDM. However, a dose-response effect was not clearly observed but rather a threshold effect below 95 nmol/L, especially in the model that addressed the low prevalence of cyclosporine use by restricting to patients treated with tacrolimus. More than two-thirds of all PTDM cases (69%) were diagnosed in the first six months. Sensitivity analyses showed that lower 25(OH)D was consistently associated with higher risk of PTDM, but this was not statistically significant for all analyses. We did not observe any significant effect measure modification in the relationship between 25(OH)D and PTDM across pre-specified subgroups.

The observed median baseline 25(OH)D level of 66 nmol/L in this study cohort was higher than reported numbers in other dialysis populations.^6,10^ Based on this finding, we also analyzed our data using cutoffs that were defined by statistical quartiles to examine whether there was still a graded effect between the relationship of vitamin D and PTDM. In our fully adjusted model, when assessed as a categorical variable using statistical quartiles, lower groups vitamin D was significantly associated with higher PTDM. Patients who had baseline 25(OH)D levels below 95 nmol/L had approximately a two-fold increase in the risk of developing PTDM compared to patients with 25(OH)D levels above 95 nmol/L. This finding was consistent in our sensitivity analyses. These results suggest that the definition of acceptable vitamin D levels among a population of kidney transplant candidates may need to be reconsidered in the context of PTDM risk.

Our main findings were supported by experimental studies that have demonstrated a link between vitamin D and impaired glucose metabolism as well as insulin secretion in animal models. Vitamin D deficient rats showed an inhibition of pancreatic insulin secretion^11^ and this was rescued with vitamin D treatment.^12^ Studies have also suggested that normal insulin secretion was dependent on vitamin D. Bland et al. showed that pancreatic ß-cells express 1-α-hydroxylase enzyme to convert 25(OH)D into the active 1,25(OH)_2_D metabolite.^13^ Furthermore, it has also been found that vitamin D receptors were present on pancreatic ß-cells for activation of vitamin D, thereby increasing insulin responsiveness to glucose levels.^14^ An alternative proposed mechanism was that vitamin D plays a role in regulating intracellular calcium levels by mobilizing calcium storage in the phospholipid pathway.^14^ Since insulin secretion was a calcium-dependent process, modulation of calcium storages by vitamin D was necessary for insulin signal transduction^15^ and glucose transporter-4 activity.^16^ These findings have also been replicated in population studies, showing that insulin sensitivity and ß-cell function were impaired with lower vitamin D levels.^17^

Our results also corroborate findings from the general population, which have shown that low serum 25(OH)D levels were associated with a risk of type 2 diabetes mellitus (T2DM). Observational studies in non-North American populations^18,19^ have reported that the highest vitamin D group has a 26 to 78% lower risk of developing T2DM than the lowest vitamin D group. In addition, a meta-analysis of 22 observational studies with 98 190 patients showed a 22% increased risk of T2DM with each 25.0 nmol/L decrease in 25(OH)D concentration, though the authors noted significant heterogeneity across studies.^20^ These results are in contrast to studies that have found no protective effect of vitamin D on the risk of developing T2DM.^21^ A Mendelian randomization study, which addressed the patient inter-individual variability of circulating 25(OH)D concentration due to 4 genetic variants of vitamin D, pooled data from 104 488 patients of European descent and concluded that there was unlikely to be a causal effect between 25(OH)D levels and T2DM.^20^ Evidence from large randomized controlled trials in the Women’s Health Initiative^22^ and the RECORD^23^ trial with 33 951 and 5292 patients, respectively, suggested that vitamin D supplementation does not reduce the risk of T2DM. The absence of an effect could be due to the low dose of vitamin D that was supplied (less than 800 IU per day). Data from The Nurses’ Health Study showed that vitamin D and calcium supplementation reduced the risk of T2DM, but not vitamin D alone.^24^ Based on the findings of these studies, it remains unclear whether supplementation of vitamin D reduces the risk of T2DM.

To our knowledge, only one other study has examined the association of 25(OH)D levels and the risk of PTDM in kidney transplant recipients. LeFur et al. studied a cohort of 444 kidney recipients in a single French center from January 2000 to December 2010 with a median 25(OH) level of 19.4 ng/ml (48.5 nmol/L).^25^ The cumulative incidence of PTDM over the first year was 13.2%. They found a significantly elevated relative hazard for PTDM of 2.41-fold in patients who were 25(OH)D deficient (≤ 10 ng/ml or 25 nmol/L) vs. replete (≥ 30 ng/ml or 75 nmol/L).^25^ The general consistency in the results of LeFur et al. and the current study was seen despite differences in the 25(OH)D distributions, ascertainment of PTDM events (American Diabetes Association criteria in the current study vs. need for hypoglycemic therapy in LeFur et al.), and cumulative incidence of PTDM. This further corroborate the association between 25(OH)D and PTDM risk.

The risk of hypovitaminosis D is substantial in kidney recipients, as patients are advised to avoid sun exposure and wear sun block to mitigate the heightened risk of skin malignancies associated with immunotherapy.^26^ When coupled with an under-appreciation of the high burden of low vitamin D levels and the under-utilization of vitamin D supplements, it is not surprising that vitamin D deficiency is highly prevalent in the kidney transplant population. Moreover, population studies have suggested that hypovitaminosis D was associated with increased risks of graft rejection, graft failure, and all-cause mortality posttransplant.^10^ Altogether, this suggests that there may be a need to propose stronger interventions to combat vitamin D insufficiency and deficiency.

Our study has several strengths including a large, multi-center cohort of 442 patients with more than 823 person-years of follow-up, standardized exposure and outcome ascertainment, application of multivariable modeling strategies to account for potential confounders, and the conduct of sensitivity analyses to examine the robustness of the main results. Despite the strengths of the study, interpretation of our study results should consider the following limitations. First, we did not include donor information other than donor type in the adjusted models. There is insufficient evidence to believe that donor characteristics would confound recipient baseline 25(OH)D levels and PTDM in the postulated causal relationship. Second, despite the size of the cohort, the number of patients in the lowest 25(OH)D group (< 40 nmol/L) was small (n = 97) compared to the other 25(OH)D groups and this may have affected the precision of our estimates. Third, this study may have been subject to selection bias since patients without available patient serum samples were excluded from the cohort, particularly patients who were transplanted from 2005 to 2008. Lastly, we obtained serum samples from recipients within a year of their transplant date, and not at the time of transplant. Fluctuations in 25(OH)D over this time could potentially affect a patient’s risk of PTDM. Our sensitivity analysis of assessing samples acquired within 3 months pre-transplant showed that the findings were consistent. Moreover, the reliability of one serum measure of 25(OH)D may be sufficient to estimate 25(OH)D levels across a span of several months after accounting for seasonal variation.

## Conclusion

This study demonstrated that 25(OH)D status at baseline was a significant independent risk factor for PTDM in kidney transplant recipients. Further research is needed to understand the relationship between vitamin D and PTDM, the mechanisms that underlie this association, and to determine if appropriate levels of vitamin D supplementation may be beneficial in reducing the risk of PTDM.

## Supplemental Material

Supplemental Material, sj-docx-1-pit-10.1177_15269248211002796 - Vitamin D Levels and the Risk of Posttransplant Diabetes Mellitus After Kidney Transplantation Click here for additional data file. Supplemental Material, sj-docx-1-pit-10.1177_15269248211002796 for Vitamin D Levels and the Risk of Posttransplant Diabetes Mellitus After Kidney Transplantation by Kevin Quach, Monica Abdelmasih, Pei Xuan Chen, Yanhong Li, Olusegun Famure, Michelle Nash, Ramesh Prasad, Bruce A. Perkins, Paul M. Yip and S. Joseph Kim in Progress in Transplantation

Supplemental Material, sj-docx-2-pit-10.1177_15269248211002796 - Vitamin D Levels and the Risk of Posttransplant Diabetes Mellitus After Kidney Transplantation Click here for additional data file. Supplemental Material, sj-docx-2-pit-10.1177_15269248211002796 for Vitamin D Levels and the Risk of Posttransplant Diabetes Mellitus After Kidney Transplantation by Kevin Quach, Monica Abdelmasih, Pei Xuan Chen, Yanhong Li, Olusegun Famure, Michelle Nash, Ramesh Prasad, Bruce A. Perkins, Paul M. Yip and S. Joseph Kim in Progress in Transplantation

Supplemental Material, sj-docx-3-pit-10.1177_15269248211002796 - Vitamin D Levels and the Risk of Posttransplant Diabetes Mellitus After Kidney Transplantation Click here for additional data file. Supplemental Material, sj-docx-3-pit-10.1177_15269248211002796 for Vitamin D Levels and the Risk of Posttransplant Diabetes Mellitus After Kidney Transplantation by Kevin Quach, Monica Abdelmasih, Pei Xuan Chen, Yanhong Li, Olusegun Famure, Michelle Nash, Ramesh Prasad, Bruce A. Perkins, Paul M. Yip and S. Joseph Kim in Progress in Transplantation
