# Supplementary material for: Vitamin D Levels and the Risk of Posttransplant Diabetes Mellitus After Kidney Transplantation
Source: Prog Transplant. 2021 Apr 1;31(2):133–41. doi: 10.1177/15269248211002796 (PMC8182337; doi:10.1177/15269248211002796)
Supplement: Supplemental Material, sj-docx-1-pit-10.1177_15269248211002796 - Vitamin D Levels and the Risk of Posttransplant Diabetes Mellitus After Kidney Transplantation [file sj-docx-1-pit-10.1177_15269248211002796.docx]

**Supplemental Table 3.** Percentage of missing values in each variable in the multivariable Cox models

| **Variable** | **Missing** | **Total** | **Percentage Missing (%)** |
| --- | --- | --- | --- |
| Vitamin D | 0 | 442 | 0 |
| Recipient age | 0 | 442 | 0 |
| Recipient sex | 0 | 442 | 0 |
| Recipient race | 49 | 442 | 11.09 |
| Recipient BMI | 6 | 442 | 1.36 |
| Time on dialysis before transplant | 23 | 442 | 5.2 |
| Recipient cause of ESRD | 0 | 442 | 0 |
| Donor type | 0 | 442 | 0 |
| Type of induction | 14 | 442 | 3.17 |
| Prednisone at discharge | 1 | 442 | 0.23 |
| Type of CNI | 6 | 442 | 1.36 |
| Albumin | 0 | 442 | 0 |
| Calcium | 0 | 442 | 0 |
| Parathyroid hormone | 2 | 442 | 0.45 |
| Season | 0 | 442 | 0 |
| Transplant era | 0 | 442 | 0 |
