# Supplementary material for: Vitamin D Levels and the Risk of Posttransplant Diabetes Mellitus After Kidney Transplantation
Source: Prog Transplant. 2021 Apr 1;31(2):133–41. doi: 10.1177/15269248211002796 (PMC8182337; doi:10.1177/15269248211002796)
Supplement: Supplemental Material, sj-docx-2-pit-10.1177_15269248211002796 - Vitamin D Levels and the Risk of Posttransplant Diabetes Mellitus After Kidney Transplantation [file sj-docx-2-pit-10.1177_15269248211002796.docx]

**Supplemental Table 1.** Association of Vitamin D Level and Posttransplant Diabetes Excluding Events Within the First 1- or 3-Months Post-Transplant

| **Vitamin D (nmoL/L)** | **Hazard Ratio (95% C.I.) of PTDM** | | | |
| --- | --- | --- | --- | --- |
|  | **Exclude PTDM events within the first month post-transplant** | ***P* value** | **Exclude PTDM events within the first 3-months post-transplant** | ***P* value** |
| Continuous (per 10 nmol/L decrease) | 1.06 (1.00, 1.14) | 0.07 | 1.13 (1.02, 1.25) | 0.02 |
| Categorical |  | |  | |
| **NKF-KDOQI** | | | | |
| Replete: >75.0 | referent | | referent | |
| Insufficient: 40.0-74.9 | 1.57 (0.91, 2.72) | 0.11 | 2.12 (0.95, 4.72) | 0.07 |
| Deficient: <40.0 | 1.27 (0.67, 2.42) | 0.47 | 2.20 (0.91, 5.29) | 0.08 |
| **Statistical quartile** | | | | |
| >95.0 | referent | | referent | |
| 65.0-94.9 | 1.83 (0.92, 3.67) | 0.09 | 2.10 (0.76, 5.81) | 0.15 |
| 42.0-64.9 | 2.09 (1.04, 4.23) | 0.04 | 2.23 (0.80, 6.21) | 0.12 |
| <42.0 | 1.73 (0.84, 3.58) | 0.14 | 2.62 (0.95, 7.20) | 0.06 |
