# Supplementary material for: Vitamin D Levels and the Risk of Posttransplant Diabetes Mellitus After Kidney Transplantation
Source: Prog Transplant. 2021 Apr 1;31(2):133–41. doi: 10.1177/15269248211002796 (PMC8182337; doi:10.1177/15269248211002796)
Supplement: Supplemental Material, sj-docx-3-pit-10.1177_15269248211002796 - Vitamin D Levels and the Risk of Posttransplant Diabetes Mellitus After Kidney Transplantation [file sj-docx-3-pit-10.1177_15269248211002796.docx]

**Supplemental Table 2. Hazard ratios for Cox model covariates**

| **Patient Characteristics** | **Hazard Ratio (95% C.I.)** | ***P* value** | **Hazard Ratio (95% C.I.)** | ***P* value** | **Hazard Ratio (95% C.I.)** | ***P* value** |
| --- | --- | --- | --- | --- | --- | --- |
| Continuous Vitamin D (per 10 nmoL/L decreases) | 1.06 (1.01, 1.13) | 0.03 |  |  |  |  |
| Categorical Vitamin D NKF-KDOQI |  |  |  |  |  |  |
| >75.0 |  |  | referent |  |  |  |
| 40.0-74.9 |  |  | 1.52 (0.96, 2.39) | 0.07 |  |  |
| <40.0 |  |  | 1.30 (0.76, 2.23) | 0.34 |  |  |
| Categorical Vitamin D Statistical Quartile |  |  |  |  |  |  |
| >95.0 |  |  |  |  | referent |  |
| 65.0-94.9 |  |  |  |  | 1.85 (1.03, 3.32) | 0.04 |
| 42.0-64.9 |  |  |  |  | 2.01 (1.12, 3.60) | 0.02 |
| <42.0 |  |  |  |  | 1.77 (0.96, 3.25) | 0.07 |
| Recipient age at transplant (every 1 year increase) | 1.05 (1.03, 1.07) | < 0.001 | 1.05 (1.03, 1.07) | < 0.001 | 1.05 (1.03, 1.06) | < 0.001 |
| Recipient sex (Female vs. Male) | 1.18 (0.81, 1.72) | 0.39 | 1.16 (0.80, 1.70) | 0.43 | 1.17 (0.80, 1.71) | 0.41 |
| Recipient race (Non-white vs. White) | 0.97 (0.62, 1.53) | 0.90 | 0.88 (0.56, 1.38) | 0.58 | 0.89 (0.57, 1.41) | 0.63 |
| Mean recipient BMI (every 1 kg/m^2^ increases) | 1.06 (1.01, 1.11) | 0.01 | 1.06 (1.01, 1.11) | 0.01 | 1.06 (1.02, 1.11) | 0.01 |
| Time on dialysis before transplant (every 1 year increase) | 0.97 (0.89, 1.05) | 0.46 | 0.97 (0.90, 1.05) | 0.49 | 0.97 (0.90, 1.06) | 0.54 |
| Cause of ESRD |  |  |  |  |  |  |
| GN | referent |  | referent |  | referent |  |
| PKD | 1.03 (0.60, 1.75) | 0.93 | 1.05 (0.61, 1.78) | 0.87 | 1.05 (0.62, 1.79) | 0.85 |
| Other | 0.65 (0.42, 1.01) | 0.05 | 0.65 (0.42, 1.01) | 0.06 | 0.64 (0.41, 1.00) | 0.05 |
| Donor type (Living vs. Deceased) | 0.50 (0.29, 0.86) | 0.01 | 0.50 (0.29, 0.86) | 0.01 | 0.50 (0.28, 0.87) | 0.01 |
| Type of induction (IL2RB vs. Anti-lymphocyte product) | 1.29 (0.84, 1.96) | 0.24 | 1.24 (0.81, 1.88) | 0.32 | 1.24 (0.81, 1.90) | 0.33 |
| Type of CNI (Cyclosporine vs. Tacrolimus) | 0.82 (0.35, 1.92) | 0.65 | 0.80 (0.34, 1.87) | 0.60 | 0.81 (0.34, 1.91) | 0.63 |
| Prednisone at discharge date (Yes vs. No) | 1.97 (1.22, 3.19) | 0.01 | 1.96 (1.21, 3.18) | 0.01 | 1.95 (1.20, 3.17) | 0.01 |
| Albumin (every 1 unit increase) | 1.00 (0.95, 1.05) | 0.96 | 0.99 (0.94, 1.04) | 0.68 | 1.00 (0.95, 1.05) | 0.95 |
| Calcium (every 1 unit increase) | 1.04 (0.54, 2.02) | 0.90 | 1.03 (0.53, 2.02) | 0.92 | 0.99 (0.50, 1.99) | 0.99 |
| Parathyroid hormone (PTH) (every 1 unit increase) | 1.00 (1.00, 1.01) | 0.41 | 1.00 (1.00, 1.01) | 0.53 | 1.00 (1.00, 1.01) | 0.43 |
| Season |  |  |  |  |  |  |
| Summer | referent |  | referent |  | referent |  |
| Spring | 1.13 (0.62, 2.06) | 0.68 | 1.14 (0.62, 2.08) | 0.67 | 1 (0, 0)* | NA |
| Fall | 0.61 (0.35, 1.07) | 0.08 | 0.62 (0.35, 1.10) | 0.10 | 1 (0, 0)* | NA |
| Winter | 0.87 (0.48, 1.60) | 0.66 | 0.87 (0.48, 1.60) | 0.66 | 1 (0, 0)* | NA |
| Transplant era |  |  |  |  |  |  |
| 2005 – 2008 | referent |  | referent |  | referent |  |
| 2009 | 0.26 (0.16, 0.44) | < 0.001 | 0.27 (0.16, 0.44) | < 0.001 | 0.26 (0.16, 0.43) | < 0.001 |
| 2010 | 0.37 (0.22, 0.61) | < 0.001 | 0.37 (0.22, 0.61) | < 0.001 | 0.35 (0.21, 0.58) | < 0.001 |

* Cox proportional hazards model was stratified by season thus coefficients for season were not estimable.
